# Association of Radiotherapy With Survival in Women Treated for Ductal Carcinoma In Situ With Lumpectomy or Mastectomy

**DOI:** 10.1001/jamanetworkopen.2018.1100

**Published:** 2018-08-10

**Authors:** Vasily Giannakeas, Victoria Sopik, Steven A. Narod

**Affiliations:** 1 Women’s College Research Institute, Toronto, Ontario, Canada; 2 Dalla Lana School of Public Health, University of Toronto, Toronto, Ontario, Canada; 3 Institute of Medical Science, University of Toronto, Toronto, Ontario, Canada

## Abstract

**Importance:**

Patients with ductal carcinoma in situ (DCIS) are treated with radiotherapy to reduce their risk of local invasive recurrence after breast-conserving surgery. However, the association of radiotherapy with breast cancer survival in patients with DCIS has not yet been clearly established.

**Objective:**

To determine the extent to which radiotherapy is associated with reduced risk of breast cancer mortality in a large cohort of patients treated for DCIS, using a propensity score–based matching approach.

**Design, Setting, and Participants:**

This cohort study of women who had first primary DCIS diagnosed between 1998 and 2014 used data from the Surveillance, Epidemiology, and End Results 18 registries database. Information on age and year of diagnosis, ethnicity, income, tumor size, tumor grade, estrogen receptor status, all treatments (surgery and radiation), and outcomes (invasive local recurrence and death from breast cancer) was abstracted for 140 366 women diagnosed with first primary DCIS. Three separate comparisons were performed using 1:1 matching: lumpectomy with radiation vs lumpectomy alone; lumpectomy alone vs mastectomy; and lumpectomy with radiation vs mastectomy.

**Exposures:**

Use of radiotherapy and/or extent of surgery.

**Main Outcomes and Measures:**

Crude and adjusted 15-year breast cancer–specific mortality.

**Results:**

Of the 140 366 patients with DCIS in the cohort (109 712 [78.2%] white; mean [SD] age, 58.8 [12.3] years), 35 070 (25.0%) were treated with lumpectomy alone, 65 301 (46.5%) were treated with lumpectomy and radiotherapy, and 39 995 (28.5%) were treated with mastectomy. The actuarial 15-year breast cancer mortality rate was 2.33% for patients treated with lumpectomy alone, 1.74% for patients treated with lumpectomy and radiation, and 2.26% for patients treated with mastectomy. The adjusted hazard ratios for death were 0.77 (95% CI, 0.67-0.88) for lumpectomy and radiotherapy vs lumpectomy alone (29 465 propensity-matched pairs), 0.91 (95% CI, 0.78-1.05) for mastectomy alone vs lumpectomy alone (20 832 propensity-matched pairs), and 0.75 (95% CI, 0.65-0.87) for lumpectomy and radiotherapy vs mastectomy (29 865 propensity-matched pairs).

**Conclusions and Relevance:**

In patients with DCIS, treatment with lumpectomy and radiotherapy was associated with a significant reduction in breast cancer mortality compared with either lumpectomy alone or mastectomy alone. This suggests that the survival benefit of radiation is likely not due to local control, but rather to systemic effects.

## Key Points

**Question:**

Is adjuvant radiation associated with a reduction in breast cancer mortality in patients treated for ductal carcinoma in situ?

**Findings:**

Using a matched approach in a large cohort of patients treated for ductal carcinoma in situ, treatment with lumpectomy and radiotherapy was associated with a significantly reduced risk of breast cancer–specific mortality compared with treatment with lumpectomy alone (hazard ratio, 0.77; 95% CI, 0.67-0.88) or mastectomy alone (hazard ratio, 0.75; 95% CI, 0.65-0.87).

**Meaning:**

Adjuvant radiation is associated with a small but significant breast cancer survival benefit in patients with ductal carcinoma in situ that cannot be accounted for by enhancing local control.

## Teaser

This cohort study uses data from the Surveillance, Epidemiology, and End Results (SEER) 18 database to compare rates of survival among women with ductal carcinoma in situ (DCIS) who were treated with radiotherapy after lumpectomy vs lumpectomy or mastectomy alone.

## Introduction

*Ductal carcinoma in situ* (DCIS) refers to the histologic appearance of cancer cells within the breast ductule and/or lobule without evidence of cancer present beyond the basement membrane.^1^ This condition is generally identified in asymptomatic women in the context of screening mammography, and the incidence of DCIS in a population closely mirrors the extent of mammographic screening.^2^ In about 15% of cases of DCIS treated with breast-conserving surgery, the woman will experience an in-breast invasive recurrence in the same breast within 15 years.^3^ In about 6% of cases, women with DCIS will develop a contralateral invasive breast cancer within 15 years.^3^ In about 3% of cases, women with DCIS will die of breast cancer within 15 years.^4^ The risk of death from breast cancer increases greatly after an in-breast invasive recurrence; however, about 50% of women who die of breast cancer after DCIS have no record of an invasive recurrence.^4^

The dual goals of treatment are to prevent invasive local recurrence and to reduce death from breast cancer. The risk of death from breast cancer for patients with DCIS is approximately the same for women treated with mastectomy as it is for those treated with lumpectomy without radiotherapy, despite the fact that women in the latter group experience many more local recurrences.^3,4,5,6,7^ There is emerging evidence that, after a diagnosis of DCIS, the addition of radiotherapy to lumpectomy reduces the risk of death from breast cancer (as well as reducing the risk of local recurrence).^8^ Because of the low mortality associated with DCIS, it is difficult to study deaths from DCIS using small cohort studies or randomized trials. As a result, most clinical trials have been designed to study local recurrence. It is challenging to study mortality because the effect sizes are small and it is necessary to compare groups of women with similar risk profiles, ie, hazard ratios must be adjusted for variations in both pathologic features and treatments. We conducted a historical cohort study of women with pure DCIS (ie, without microinvasion) using the Surveillance, Epidemiology, and End Results (SEER) database. We extracted data on age and year of diagnosis, tumor size, tumor grade, treatments (surgery and radiation), and outcomes (local invasive recurrence, contralateral invasive breast cancer, and death from breast cancer). We sought to measure the extent to which radiotherapy is associated with a reduced risk of breast cancer death in this cohort of women and to identify subgroups of women who might benefit from radiotherapy the most.

## Methods

We used SEER*Stat statistical software version 8.3.4 to conduct a case-listing session and retrieved all cases of first primary DCIS (stage 0) diagnosed between 1998 and 2014 in the SEER 18 registries research database (November 2016 submission). We selected all cases with the American Joint Committee on Cancer primary tumor classification Tis (carcinoma in situ; no evidence of an invasive component). Among the cases classified as Tis, we excluded those associated with lobular carcinoma in situ, nonepithelial histologies, Paget disease of the nipple, or diffuse DCIS. We also excluded cases with unknown laterality, unknown or no surgical intervention on the primary tumor, and unknown radiation treatment status. Information on exclusions is provided in eTable 1 in the Supplement. Because patients cannot be identified, the research ethics board of the Women’s College Hospital exempted this study from review, and patient informed consent was not required. This article follows the Strengthening the Reporting of Observational Studies in Epidemiology (STROBE) reporting guideline for cohort studies.

For each case, we retrieved information on the year of breast cancer diagnosis, age at diagnosis, ethnicity, household income, tumor laterality, tumor size, tumor grade, estrogen receptor (ER) status, progesterone receptor status, use of radiotherapy, use of chemotherapy, type of surgery, and cause of death. We assessed the vital status at the time of last follow-up. We extracted the information on survival time from the variable *survival time months*. The SEER*Stat program estimates survival time by subtracting the date of diagnosis from the date of last contact (the study cutoff).

For each case we linked all additional cancer events that followed the DCIS diagnosis. Ipsilateral invasive recurrence was defined as the earliest new primary record that was an invasive breast cancer (stage I to IV) that occurred in the same breast as the DCIS. We retrieved all tumor characteristics and treatments for the ipsilateral invasive recurrence. We defined 3 time intervals: time from DCIS to end of follow-up, time from DCIS to ipsilateral invasive recurrence, and time from DCIS to contralateral invasive breast cancer. Outcome events were breast cancer–specific mortality, ipsilateral invasive recurrence, and contralateral invasive breast cancer, respectively.

Study participants were categorized into 3 groups: mastectomy, lumpectomy without radiation, and lumpectomy with radiation. The groups were compared for a range of demographic, pathologic, and treatment variables and differences were evaluated using standardized differences.

### Matching

We conducted 3 separate cohort comparisons using 1:1 matching: lumpectomy with radiation vs lumpectomy without radiation, lumpectomy without radiation vs mastectomy, and lumpectomy with radiation vs mastectomy. In each analysis, patients were matched on year of diagnosis (same year), age at diagnosis (within 2 years), tumor grade (I, II, III, or IV), ER status (positive, negative, or unknown), and propensity score. The propensity score took into account ethnicity, household income, tumor size, and progesterone receptor status. Caliper matching was done by matching participants who were within 0.2 times the standard deviation of their propensity score.^9^ A standardized difference of greater than 0.1 was considered a meaningful imbalance between comparison groups.^10^ Variable distributions for the matched cohorts are available in eTables 2, 3, and 4 in the Supplement.

### Statistical Analysis

We estimated the crude cumulative breast cancer–specific mortality rates for the 3 treatment-matched subgroups using the Kaplan-Meier method. We then estimated the crude rates for invasive local recurrence (from the date of diagnosis of DCIS to the date of ipsilateral invasive recurrence for the 3 treatment groups).

Hazard ratios (HRs) were calculated using the Cox proportional hazards model in SAS statistical software, version 9.4 (SAS Institute Inc). Patients were followed up from the date of DCIS until the outcome of interest, the end of follow-up, death from another cause, or loss to follow-up. Adjusted HRs were generated using a Cox proportional hazards model on the matched subgroups.

Among all participants treated with lumpectomy, we conducted subgroup comparisons by age, ethnicity, ER status, tumor grade, and tumor size (using inverse probability of treatment weighting) to determine the extent to which radiation was associated with decreased risk of death in these various subgroups. Stabilized inverse probability of treatment–weighted estimates were truncated at the 1st and 99th percentile.^10,11^

Breast cancer–specific mortality hazard rates were calculated for each year following DCIS diagnosis. We compared hazard rates for the entire 15-year interval assuming a proportional hazard and then for three 5-year subintervals (0-5 years, 5-10 years, and 10-15 years after diagnosis). In this analysis, the hazard rate was permitted to vary between intervals but was proportional within a given interval.

A log-rank test was used to compare differences across groups with the Kaplan-Meier method. We generated 95% confidence limits for all HRs in the analysis. All *P* values were 2-tailed and statistically significant at a level of .05 or less.

## Results

Among the 140 366 patients with DCIS in the cohort (109 712 [78.2%] white; mean [SD] age, 58.8 [12.3] years), 100 371 patients (71.5%) were treated with lumpectomy (35 070 [25.0%] with lumpectomy alone and 65 301 [46.5%] with lumpectomy and radiotherapy) and 39 995 patients (28.5%) were treated with mastectomy (Table 1). The patients treated with mastectomy were slightly younger on average than those treated with lumpectomy (mean [SD] age, 56.5 [12.6] years vs 59.8 [12.0] years). The likelihood of having a mastectomy increased with tumor size and with tumor grade.

**Table 1.** Baseline Characteristics of All Patients With Ductal Carcinoma In Situ, According to Treatment Group

Value	No. (%)				*P* Value^a^
	Overall	Lumpectomy Alone	Lumpectomy Plus Radiotherapy	Mastectomy	
Patients	140 366 (100)	35 070 (25.0)	65 301 (46.5)	39 995 (28.5)	
Year of diagnosis					
1998-2004	47 675 (34.0)	13 619 (38.8)	20 343 (31.2)	13 713 (34.3)	<.001
2005-2009	45 502 (32.4)	10 923 (31.1)	21 957 (33.6)	12 622 (31.6)	
2010-2014	47 189 (33.6)	10 528 (30.0)	23 001 (35.2)	13 660 (34.2)	
Age at diagnosis, y					
Mean (SD)	58.8 (12.3)	62.1 (13.2)	58.5 (11.1)	56.5 (12.6)	<.001
Median (IQR)	58.0 (49.0-68.0)	61.0 (52.0-72.0)	58.0 (50.0-67.0)	55.0 (47.0-66.0)	<.001
<40	4657 (3.3)	780 (2.2)	1414 (2.2)	2463 (6.2)	<.001
40-49	31 047 (22.1)	6114 (17.4)	14 014 (21.5)	10 919 (27.3)	
50-59	40 338 (28.7)	8947 (25.5)	20 277 (31.1)	11 114 (27.8)	
60-69	34 504 (24.6)	8151 (23.2)	17 856 (27.3)	8497 (21.2)	
70-79	22 116 (15.8)	7135 (20.3)	9733 (14.9)	5248 (13.1)	
≥80	7704 (5.5)	3943 (11.2)	2007 (3.1)	1754 (4.4)	
Ethnicity					
White	109 712 (78.2)	27 765 (79.2)	51 261 (78.5)	30 686 (76.7)	<.001
Black	14 904 (10.6)	3542 (10.1)	6910 (10.6)	4452 (11.1)	
East Asian	5983 (4.3)	1336 (3.8)	2915 (4.5)	1732 (4.3)	
Southeast Asian	5364 (3.8)	1183 (3.4)	2412 (3.7)	1769 (4.4)	
Other or unknown	4403 (3.1)	1244 (3.5)	1803 (2.8)	1356 (3.4)	
Annual household income, $					
<30 000	38 844 (27.7)	8282 (23.6)	18 426 (28.2)	12 136 (30.3)	<.001
30 000-34 999	35 561 (25.3)	11 165 (31.8)	14 559 (22.3)	9837 (24.6)	
35 000-39 999	27 795 (19.8)	6210 (17.7)	13 752 (21.1)	7833 (19.6)	
≥40 000	38 153 (27.2)	9408 (26.8)	18 561 (28.4)	10 184 (25.5)	
Unknown	13 (0.0)	5 (0.0)	3 (0.0)	5 (0.0)	
Tumor grade					
I	16 620 (11.8)	6198 (17.7)	7166 (11.0)	3256 (8.1)	<.001
II	48 404 (34.5)	13 259 (37.8)	22 859 (35.0)	12 286 (30.7)	
III or IV	53 597 (38.2)	8696 (24.8)	26 276 (40.2)	18 625 (46.6)	
Unknown	21 745 (15.5)	6917 (19.7)	9000 (13.8)	5828 (14.6)	
Tumor size, cm					
Mean (SD)	1.7 (2.1)	1.3 (2.0)	1.4 (1.5)	2.6 (2.7)	<.001
Median (IQR)	1.1 (0.6-2.0)	0.8 (0.5-1.5)	1.0 (0.5-1.7)	1.8 (1.0-3.5)	<.001
<1.0	42 267 (30.1)	12 861 (36.7)	22 381 (34.3)	7025 (17.6)	<.001
1.0-1.9	28 500 (20.3)	5814 (16.6)	15 208 (23.3)	7478 (18.7)	
2.0-2.9	12 434 (8.9)	2094 (6.0)	5700 (8.7)	4640 (11.6)	
3.0-4.9	9263 (6.6)	1385 (3.9)	3450 (5.3)	4428 (11.1)	
≥5.0	6823 (4.9)	874 (2.5)	1421 (2.2)	4528 (11.3)	
Unknown	41 079 (29.3)	12 042 (34.3)	17 141 (26.2)	11 896 (29.7)	
Estrogen receptor status					
Negative	13 823 (9.8)	2021 (5.8)	6576 (10.1)	5226 (13.1)	<.001
Positive	77 023 (54.9)	17 050 (48.6)	39 242 (60.1)	20 731 (51.8)	
Unknown	49 520 (35.3)	15 999 (45.6)	19 483 (29.8)	14 038 (35.1)	
Progesterone receptor status					
Negative	21 482 (15.3)	3399 (9.7)	10 497 (16.1)	7586 (19.0)	<.001
Positive	63 877 (45.5)	14 364 (41.0)	32 690 (50.1)	16 823 (42.1)	
Unknown	55 007 (39.2)	17 307 (49.3)	22 114 (33.9)	15 586 (39.0)	

Abbreviation: IQR, interquartile range.

^a^ Variables statistically different across all treatment combinations. We used χ^2^ tests for categorical variables and *t* tests and Mann-Whitney tests for continuous variables.

Among the patients treated with lumpectomy, 65 301 (65%) received radiotherapy and 35 070 (35%) did not. Among these patients, those who received radiotherapy were on average 3.6 years younger than those who did not (mean [SD] age, 58.5 [11.1] years vs 62.1 [13.2] years) (Table 1). The use of radiotherapy also increased with increasing tumor grade. Radiotherapy was less commonly used for women with cancers of less than 1 cm (64%) than for women with larger cancers (72%).

For all participants combined, the cumulative mortality from breast cancer at 15 years was 2.03% (annual rates provided in eTable 5 in the Supplement). The risk was 2.26% for participants treated with mastectomy and 1.94% for participants treated with lumpectomy. The actuarial 15-year mortality rate for women who had a mastectomy (2.26%) was similar to the rate for women who had lumpectomy without radiotherapy (2.33%). The adjusted HR for death for mastectomy vs lumpectomy alone (based on 20 832 propensity-matched pairs) was 0.91 (95% CI, 0.78-1.05) (Table 2; eFigure 1 in the Supplement).

**Table 2.** Hazard Ratios Associated With Radiation and Extent of Surgery in 1:1 Propensity-Matched Subgroups

Comparison	Hazard Ratio (95% CI)	*P* Value
Lumpectomy plus radiotherapy vs lumpectomy alone	0.77 (0.67-0.88)	<.001
Mastectomy vs lumpectomy alone	0.91 (0.78-1.05)	.20
Lumpectomy plus radiotherapy vs mastectomy	0.75 (0.65-0.87)	<.001

Among patients treated with lumpectomy, the actuarial 15-year mortality rate was 25% less for those who received radiotherapy than for those who did not (1.74% vs 2.33%). The adjusted HR associated with radiotherapy (based on 29 465 propensity-matched pairs) was 0.77 (95% CI, 0.67-0.88; *P* < .001) (Table 2 and the Figure). The adjusted HR for death associated with lumpectomy and radiotherapy vs mastectomy (based on 29 865 propensity-matched pairs) was 0.75 (95% CI, 0.65-0.87; *P* < .001). The results of the adjusted analysis did not change substantially when competing risks of death were considered in the model (model 2 in eTable 6 in the Supplement) or when inverse probability of treatment weighting was used to compare treatment groups (model 3 in eTable 6 in the Supplement). In the matched lumpectomy cohort, radiotherapy was associated with an absolute reduction in local recurrences of 2.82% (eTable 7 and eFigure 2 in the Supplement) and a reduction in deaths from breast cancer of 0.27% (eTable 7 in the Supplement; Figure). In the matched comparison of patients treated with lumpectomy and radiation vs mastectomy, mastectomy was associated with an absolute reduction in local recurrences of 4.31% (eTable 8 and eFigure 3 in the Supplement) and an absolute increase in breast cancer deaths of 0.28% (eTable 8 and eFigure 4 in the Supplement).

**Figure.**
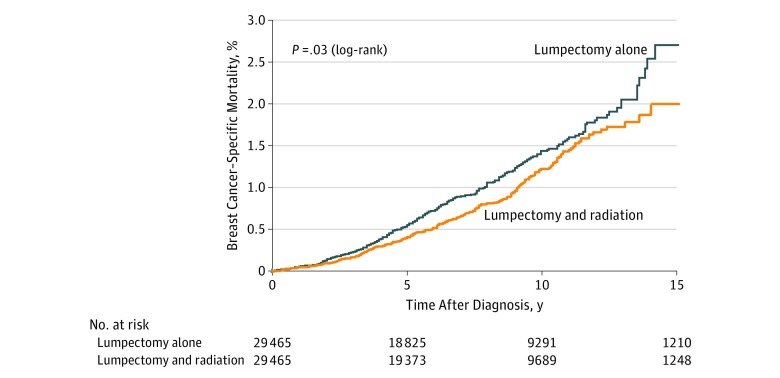
Breast Cancer–Specific Mortality After Ductal Carcinoma In Situ in Propensity-Matched Patients Treated With Lumpectomy Alone vs Lumpectomy and Radiotherapy

The protective effect of radiotherapy on mortality was measured for different subgroups of patients who underwent lumpectomy using inverse probability of treatment weighting (Table 3). The HR was 0.59 (95% CI, 0.43-0.80) for patients younger than 50 years and 0.86 (95% CI, 0.73-1.01) for patients aged 50 years and older. The HR was 0.67 (95% CI, 0.51-0.87) for patients with ER-positive cancers, 0.50 (95% CI, 0.32-0.78) for patients with ER-negative cancers, and 0.93 (95% CI, 0.77-1.13) for patients with unknown ER status. The HR was 0.69 (95% CI, 0.50-0.96) for black women and 0.83 (95% CI, 0.71-0.98) for white women. The HR was 1.00 (95% CI, 0.79-1.27) for patients with low- or intermediate-grade tumors (grade I or II) and 0.59 (95% CI, 0.47-0.75) for patients with high-grade tumors (grade III or IV).

**Table 3.** Hazard Ratios Associated With Lumpectomy and Radiotherapy vs Lumpectomy Alone for Various Patient Subgroups (Adjusted Using Inverse Probability of Treatment Weighting)

Subgroup	Comparison	Hazard Ratio (95% CI)	*P* Value
Estrogen receptor status^a^			
Positive	Lumpectomy alone	1 [Reference]	.003
	Lumpectomy plus radiotherapy	0.67 (0.51-0.87)	
Negative	Lumpectomy alone	1 [Reference]	.002
	Lumpectomy plus radiotherapy	0.50 (0.32-0.78)	
Unknown	Lumpectomy alone	1 [Reference]	.46
	Lumpectomy plus radiotherapy	0.93 (0.77-1.13)	
Age at diagnosis, y			
<40	Lumpectomy alone	1 [Reference]	.09
	Lumpectomy plus radiotherapy	0.54 (0.26-1.09)	
40-49	Lumpectomy alone	1 [Reference]	.004
	Lumpectomy plus radiotherapy	0.59 (0.42-0.84)	
50-59	Lumpectomy alone	1 [Reference]	.01
	Lumpectomy plus radiotherapy	0.68 (0.50-0.92)	
≥60	Lumpectomy alone	1 [Reference]	.29
	Lumpectomy plus radiotherapy	0.90 (0.74-1.09)	
Ethnicity			
White	Lumpectomy alone	1 [Reference]	.03
	Lumpectomy plus radiotherapy	0.83 (0.71-0.98)	
Black	Lumpectomy alone	1 [Reference]	.03
	Lumpectomy plus radiotherapy	0.69 (0.50-0.96)	
Tumor grade^a^			
I	Lumpectomy alone	1 [Reference]	.09
	Lumpectomy plus radiotherapy	1.54 (0.94-2.53)	
II	Lumpectomy alone	1 [Reference]	.31
	Lumpectomy plus radiotherapy	0.87 (0.67-1.14)	
III or IV	Lumpectomy alone	1 [Reference]	<.001
	Lumpectomy plus radiotherapy	0.59 (0.47-0.75)	
Tumor size, cm^a^			
<1.0	Lumpectomy alone	1 [Reference]	.58
	Lumpectomy plus radiotherapy	0.92 (0.68-1.24)	
1.0-1.9	Lumpectomy alone	1 [Reference]	.01
	Lumpectomy plus radiotherapy	0.68 (0.50-0.92)	
2.0-2.9	Lumpectomy alone	1 [Reference]	.24
	Lumpectomy plus radiotherapy	0.75 (0.47-1.21)	
3.0-4.9	Lumpectomy alone	1 [Reference]	.07
	Lumpectomy plus radiotherapy	0.54 (0.27-1.06)	
≥5.0	Lumpectomy alone	1 [Reference]	<.001
	Lumpectomy plus radiotherapy	0.20 (0.09-0.49)	

^a^ Global test for interaction statistically significant (*P* < .05).

In the matched cohort of patients who underwent lumpectomy, actuarial breast cancer mortality at 15 years was reduced by 0.27% with radiotherapy (from 2.05% to 1.78%). The difference was greater than this for women younger than 50 years (1.59%; from 3.06% to 1.47%), black women (0.87%; from 4.28% to 3.41%), and women with ER-negative cancers (0.57%; from 2.99% to 2.42%). On average, 370 women would need to be treated with radiotherapy to save 1 life. This count was fewer for black women (115 treated) and for women younger than 50 years (63 treated).

We sought to better characterize the time-dependent effect of the association between radiotherapy and mortality. To do this, we divided the follow-up period into three 5-year intervals and constructed interval-specific hazard rates and HRs for the matched lumpectomy cohort (Table 4). The risk of dying of breast cancer increased with time since DCIS diagnosis, from 76.4 per 100 000 person-years in the first interval to 179.1 per 100 000 person-years in the third interval. In contrast, the benefit of radiotherapy in terms of mortality reduction diminished with time; the hazard ratio was 0.71 (95% CI, 0.57-0.87) in the first interval and 1.06 (95% CI, 0.77-1.46) in the third interval. In the matched lumpectomy cohort, radiotherapy was also associated with a significant reduction in contralateral invasive breast cancers (HR, 0.91; 95% CI, 0.85-0.97).

**Table 4.** Hazard Ratios for Mortality From Breast Cancer Associated With Time Period (Time From Ductal Carcinoma In Situ Diagnosis) in Matched Patients Treated With Lumpectomy and Radiation vs Lumpectomy Alone

Time Period, y^a^	Comparison	Hazard Ratio (95% CI)	*P* Value
0-5.0	Lumpectomy alone	1 [Reference]	.001
	Lumpectomy plus radiotherapy	0.71 (0.57-0.87)	
5.1-10.0	Lumpectomy alone	1 [Reference]	.005
	Lumpectomy plus radiotherapy	0.72 (0.58-0.91)	
10.1-15.0	Lumpectomy alone	1 [Reference]	.74
	Lumpectomy plus radiotherapy	1.06 (0.77-1.46)	

^a^ Global test for interaction not statistically significant (*P* = .31).

## Discussion

Among patients with DCIS treated with lumpectomy, adjuvant radiation was associated with a 23% reduced risk of dying of breast cancer; the cumulative mortality at 15 years was 2.33% for patients with DCIS treated with lumpectomy alone and 1.74% for women treated with lumpectomy and radiotherapy (adjusted HR, 0.77; 95% CI, 0.67-0.88; *P* < .001). The relative risk reduction in mortality of 23% is substantial, but the absolute risk reduction was only 0.27%, and it is doubtful whether a benefit of this size is large enough to warrant radiotherapy. It would be necessary to treat 370 women to save 1 life. The mortality benefit for black women was larger (1 death prevented for every 115 women treated), but the small size of this difference makes it difficult to personalize treatment.

We believe that the mortality benefit is attributable to radiotherapy and not to a baseline imbalance in pathologic features or treatments; we used matching and propensity scoring to generate comparable groups (eTables 2-4 in the Supplement). Women who received radiation were younger, on average, and were more likely to have high-grade cancers than the women who did not receive radiation (Table 1), but these factors were accounted for in the matched analysis.

In the 2010 Early Breast Cancer Trialists’ Collaborative Group (EBCTCG) overview of randomized trials evaluating radiotherapy after lumpectomy in women with DCIS,^14^ radiotherapy decreased ipsilateral breast events by one-half (HR, 0.46; *P* < .001), but had no effect on breast cancer mortality (HR, 1.22; *P* > .1). Many population-based studies examining the various treatments in patients with DCIS have confirmed a reduction in local recurrences with local therapies (mastectomy vs lumpectomy and lumpectomy plus radiotherapy vs lumpectomy alone)^4,5,6,15^; however, most have reported no significant difference in breast cancer mortality.^4,5,6,7,8,15,16^

In our previous analysis of the SEER DCIS cohort,^4^ we observed a nonsignificant decrease in breast cancer mortality associated with radiotherapy after lumpectomy (adjusted HR, 0.81; 95% CI, 0.63-1.04) and a nonsignificant increase in breast cancer mortality associated with mastectomy compared with lumpectomy (adjusted HR, 1.20; 95% CI, 0.96-1.50). The current analysis examines a larger cohort of patients, and we used a propensity score–based 1:1 matching approach to compare the treatment groups. This approach eliminates the potential influence of outliers in the data set. We report HRs similar in size to those of the previous study, but which now reach statistical significance (Table 2).

In 2016, Sagara et al^8^ studied 32 144 lumpectomy-treated patients with DCIS diagnosed between 1998 and 2007 in the SEER database. In a multivariable analysis adjusted by patient age, year, patient race, tumor size, and tumor grade, the HR for death associated with radiotherapy was 0.73 (95% CI, 0.62-0.88). However, this study did not include patients treated with mastectomy; we show, to our knowledge for the first time, a survival benefit of lumpectomy plus radiotherapy compared with mastectomy (HR, 0.75; 95% CI, 0.65-0.87; *P* < .001) (Table 2).

In theory, there are various mechanisms whereby radiation might reduce mortality in patients with DCIS. If radiation exerts its effect through local control, ie, if radiation prevents local recurrences, and if local recurrences are the source of metastases, then radiation should prevent some deaths. Elsewhere we have argued against this model.^17^ It is often stated, based on results of the EBCTCG study of invasive breast cancer,^18,19^ that for every 4 local recurrences prevented, 1 death is prevented (radiation-prevented local recurrences and deaths in a ratio of 4 to 1). The association is insufficient to infer causality. In the present study, radiation after lumpectomy was associated with reductions in local recurrences by 2.82% and of deaths by 0.27%, ie, the ratio of local recurrences prevented to deaths prevented was approximately 10 to 1 (Figure; eTable 7 and eFigure 2 in the Supplement). However, we cannot infer that the decline in deaths was a consequence of avoiding recurrences because there is no direct evidence that the women who survived were those who avoided local recurrence. Moreover, in comparing the lumpectomy plus radiation cohort with the mastectomy cohort, we observed a marked decrease in local recurrences with mastectomy (4.31%), but an increase in deaths of 0.28% (eTable 8, eFigures 3 and 4 in the Supplement). If the salutary effect of radiation on mortality were effected through local control, we would expect to see the same effect (or a greater effect) with mastectomy.

Similar results have been reported for patients with invasive cancer. In the 7 trials comparing mastectomy alone with lumpectomy and radiotherapy among women with node-negative invasive breast cancer,^19^ the rate ratio for local recurrence was 0.54 (*P* < .001) and the rate ratio for breast cancer mortality was 0.98 (*P* = .80). Several studies in patients with early invasive breast cancer have shown that lumpectomy and radiotherapy combined are superior to mastectomy in terms of survival, despite being less effective in terms of local control.^20,21,22,23^

These results support our conclusion that the survival benefits of radiotherapy seen in both patients with DCIS and patients with invasive breast cancer cannot be explained by improving local control. We must seek an alternative explanation, namely that radiation to the breast acts as a systemic therapy to eradicate subclinical latent metastases. If a patient dies of breast cancer following DCIS, it is reasonable to conclude that undetected metastatic deposits were present at the time of diagnosis, and that may lead to generalized metastatic clinical disease and death. Perhaps radiation induces an immune response or activates another defense mechanism, thereby preventing the emergence or expansion of subclinical metastatic clones.^24^ Possible considerations include radiation to the blood as it circulates through the breast, radiation to stromal elements in the breast matrix, and radiation scatter to tissues beyond the breast. These areas are deserving of future study.

Support for the notion that local radiation induces systemic antitumor effects is the observation of a significant reduction in contralateral invasive breast cancers in the matched comparison of lumpectomy and radiotherapy vs lumpectomy alone (HR, 0.91; 95% CI, 0.85 to 0.97) (eFigure 5 in the Supplement). A 2017 meta-analysis of all observational and randomized studies in patients with DCIS reported an HR for radiotherapy on contralateral breast cancer of 0.95 (95% CI, 0.44-1.82).^25^ Future studies are required to more closely examine this association. This study of patients with DCIS is ideal, as fewer patients will receive chemotherapy or other systemic therapies that could affect risk.

### Limitations

Our study has several inherent limitations. It has been acknowledged that the rates of local recurrence among patients with DCIS in SEER are lower than expected, but this should not affect the mortality results. We might have misclassified some of the cases of DCIS with microinvasion as pure DCIS. In the SEER database there are currently 13 cases of pure DCIS recorded for every case of DCIS with microinvasion.^12^ Including patients with DCIS with microinvasion should not affect the protective association with radiotherapy unless women with microinvasion were less likely to receive radiotherapy than those without microinvasion. Data were missing for many individuals for key variables, including tumor size, grade, and ER status. We did not have information on tamoxifen use. It has been reported that radiotherapy is underreported in the SEER database^13^; however, we do not think that there are false-positive reports of radiotherapy and we accept that the women who reported having radiotherapy were likely to have had it. Therefore, the effect of misclassification should be small. The treatments in the study population were not assigned at random, and there is always the possibility that the decision to undergo radiotherapy was associated with other favorable prognostic factors (latent confounding) related to the tumor, demographic factors, or the treatment itself. The matching process requires the exclusion of a significant proportion of the cohort; thus, the results may not be generalizable to all patients with DCIS.

## Conclusions

Among patients with DCIS, treatment with lumpectomy and radiotherapy is associated with a significant reduction in breast cancer mortality compared with either lumpectomy alone or mastectomy. Although the clinical benefit is small, it is intriguing that radiotherapy has this effect, which appears to be attributable to systemic activity rather than local control. How exactly radiotherapy affects survival is an important question that should be explored in future studies.

## Notes

Supplement. **eTable 1.** Excluded Cases of Stage 0 Breast Cancer Identified in SEER From 1998 to 2014 **eTable 2.** Matched DCIS Patients Treated With Lumpectomy Alone Versus Lumpectomy and Radiation **eTable 3.** Matched DCIS Patients Treated With Lumpectomy Alone Versus Mastectomy **eTable 4.** Matched DCIS Patients Treated With Lumpectomy and Radiation Versus Mastectomy **eTable 5.** Breast Cancer-Specific Mortality Rates From DCIS Diagnosis for the Entire Cohort and According to Treatment Group (Mastectomy, Lumpectomy Alone, Lumpectomy and Radiotherapy) **eTable 6.** Hazard Ratios Associated With Radiation/Extent of Surgery Using Multivariate Cox Regression, Inverse Probability Treatment Weighting and 1:1 Propensity Score-Based Matching, With and Without Accounting for Competing Risks of Death Among Matched Subgroups **eTable 7.** Breast Cancer-Specific Mortality And Ipsilateral Invasive Recurrence Rates From DCIS Diagnosis Among Matched DCIS Patients Treated With Lumpectomy Alone Versus Lumpectomy and Radiation **eTable 8.** Breast Cancer-Specific Mortality and Ipsilateral Invasive Recurrence Rates From DCIS Diagnosis Among Matched DCIS Patients Treated With Mastectomy Versus Lumpectomy and Radiation **eFigure 1.** Breast Cancer-Specific Survival Post-DCIS in Propensity-Matched Patients Treated With Lumpectomy Alone vs. With Mastectomy **eFigure 2.** Ipsilateral Invasive Recurrence-Free Survival Post-DCIS in Propensity-Matched Patients Treated With Lumpectomy and Radiation vs. With Lumpectomy Alone **eFigure 3.** Breast Cancer-Specific Survival Post-DCIS in Propensity-Matched Patients Treated With Lumpectomy and Radiation vs. With Mastectomy **eFigure 4.** Ipsilateral Invasive Recurrence-Free Survival Post-DCIS in Propensity-Matched Patients Treated With Lumpectomy and Radiation vs. With Mastectomy **eFigure 5.** Contralateral Invasive Breast Cancer-Free Survival Post-DCIS in Propensity-Matched Patients Treated With Lumpectomy and Radiation vs. With Lumpectomy Alone
