# Error in Supplement

**DOI:** 10.1001/jamanetworkopen.2019.11052

**Published:** 2019-08-16

In the Original Investigation titled “Association of Radiotherapy With Survival in Women Treated for Ductal Carcinoma In Situ With Lumpectomy or Mastectomy,”^1^ published August 10, 2018, there was an error in the column heads of eTable 7 in the Supplement. In both eTable 7a and 7b, the left column head should have read “Lumpectomy alone” and the right, “Lumpectomy and radiation.” The headers were inadvertently switched. This article has been corrected.^1^

## References

[1] Giannakeas V, Sopik V, Narod SA Association of radiotherapy with survival in women treated for ductal carcinoma in situ with lumpectomy or mastectomy. JAMA Netw Open. 2018;1(4):. doi:10.1001/jamanetworkopen.2018.1100 30646103 PMC6324271

