# Supplementary material for: Association of Radiotherapy With Survival in Women Treated for Ductal Carcinoma In Situ With Lumpectomy or Mastectomy
Source: JAMA Netw Open. 2018 Aug 10;1(4):e181100. doi: 10.1001/jamanetworkopen.2018.1100 (PMC6324271; doi:10.1001/jamanetworkopen.2018.1100)
Supplement: Supplement. — eTable 1. Excluded Cases of Stage 0 Breast Cancer Identified in SEER From 1998 to 2014 eTable 2. Matched DCIS Patients Treated With Lumpectomy Alone Versus Lumpectomy and Radiation eTable 3. Matched DCIS Patients Treated With Lumpectomy Alone Versus Mastectomy eTable 4. Matched DCIS Patients Treated With Lumpectomy and Radiation Versus Mastectomy eTable 5. Breast Cancer-Specific Mortality Rates From DCIS Diagnosis for the Entire Cohort and According to Treatment Group (Mastectomy, Lumpectomy Alone, Lumpectomy and Radiotherapy) eTable 6. Hazard Ratios Associated With Radiation/Extent of Surgery Using Multivariate Cox Regression, Inverse Probability Treatment Weighting and 1:1 Propensity Score-Based Matching, With and Without Accounting for Competing Risks of Death Among Matched Subgroups eTable 7. Breast Cancer-Specific Mortality And Ipsilateral Invasive Recurrence Rates From DCIS Diagnosis Among Matched DCIS Patients Treated With Lumpectomy Alone Versus Lumpectomy and Radiation eTable 8. Breast Cancer-Specific Mortality and Ipsilateral Invasive Recurrence Rates From DCIS Diagnosis Among Matched DCIS Patients Treated With Mastectomy Versus Lumpectomy and Radiation eFigure 1. Breast Cancer-Specific Survival Post-DCIS in Propensity-Matched Patients Treated With Lumpectomy Alone vs. With Mastectomy eFigure 2. Ipsilateral Invasive Recurrence-Free Survival Post-DCIS in Propensity-Matched Patients Treated With Lumpectomy and Radiation vs. With Lumpectomy Alone eFigure 3. Breast Cancer-Specific Survival Post-DCIS in Propensity-Matched Patients Treated With Lumpectomy and Radiation vs. With Mastectomy eFigure 4. Ipsilateral Invasive Recurrence-Free Survival Post-DCIS in Propensity-Matched Patients Treated With Lumpectomy and Radiation vs. With Mastectomy eFigure 5. Contralateral Invasive Breast Cancer-Free Survival Post-DCIS in Propensity-Matched Patients Treated With Lumpectomy and Radiation vs. With Lumpectomy Alone [file jamanetwopen-1-e181100-s001.pdf]

Supplementary Online Content

Giannakeas V, Sopik V, Narod SA. Association of Radiotherapy With Survival in Women Treated for Ductal Carcinoma In Situ With Lumpectomy or Mastectomy. *JAMA Netw Open*. 2018;1(4):e181100. doi:10.1001/jamanetworkopen.2018.1100

eTable 1. Excluded Cases of Stage 0 Breast Cancer Identified in SEER From 1998 to 2014

eTable 2. Matched DCIS Patients Treated With Lumpectomy Alone Versus Lumpectomy and Radiation

eTable 3. Matched DCIS Patients Treated With Lumpectomy Alone Versus Mastectomy

eTable 4. Matched DCIS Patients Treated With Lumpectomy and Radiation Versus Mastectomy

eTable 5. Breast Cancer-Specific Mortality Rates From DCIS Diagnosis for the Entire Cohort and According to Treatment Group (Mastectomy, Lumpectomy Alone, Lumpectomy and Radiotherapy)

eTable 6. Hazard Ratios Associated With Radiation/Extent of Surgery Using Multivariate Cox Regression, Inverse Probability Treatment Weighting and 1:1 Propensity Score-Based Matching, With and Without Accounting for Competing Risks of Death Among Matched Subgroups

eTable 7. Breast Cancer-Specific Mortality And Ipsilateral Invasive Recurrence Rates From DCIS Diagnosis Among Matched DCIS Patients Treated With Lumpectomy Alone Versus Lumpectomy and Radiation

eTable 8. Breast Cancer-Specific Mortality and Ipsilateral Invasive Recurrence Rates From DCIS Diagnosis Among Matched DCIS Patients Treated With Mastectomy Versus Lumpectomy and Radiation

eFigure 1. Breast Cancer-Specific Survival Post-DCIS in Propensity-Matched Patients Treated With Lumpectomy Alone vs. With Mastectomy

eFigure 2. Ipsilateral Invasive Recurrence-Free Survival Post-DCIS in Propensity-Matched Patients Treated With Lumpectomy and Radiation vs. With Lumpectomy Alone

eFigure 3. Breast Cancer-Specific Survival Post-DCIS in Propensity-Matched Patients Treated With Lumpectomy and Radiation vs. With Mastectomy

eFigure 4. Ipsilateral Invasive Recurrence-Free Survival Post-DCIS in Propensity-Matched Patients Treated With Lumpectomy and Radiation vs. With Mastectomy

© 2018 Giannakeas V et al. *JAMA Network Open.*

eFigure 5. Contralateral Invasive Breast Cancer-Free Survival Post-DCIS in PropensityMatched Patients Treated With Lumpectomy and Radiation vs. With Lumpectomy Alone

This supplementary material has been provided by the authors to give readers additional information about their work.

© 2018 Giannakeas V et al. *JAMA Network Open.*

eTable 1. Excluded cases of stage 0 breast cancer identified in SEER from 1998 to 2014

Exclusion criteria	Frequency	Percent	Cumulative frequency	Cumulative percent
1. Prior cancers not in SEER	12,528	5.9	12,528	5.9
2. First primary is not Stage 0 breast cancer	25,519	12	38,047	17.9
3. Missing or no follow-up months	1,988	0.9	40,035	18.8
4. LCIS	23,146	10.9	63,181	29.7
5. Paget's disease	1,548	0.7	64,729	30.4
6. Unknown/invasive histology type	33	0	64,762	30.4
7. Unknown laterality	65	0	64,827	30.5
8. Did not undergo surgery	3,833	1.8	68,660	32.3
9. Unknown surgery	1,468	0.7	70,128	33
10. Unknown radiation treatment	2,213	1	72,341	34
**Eligible for study**	**140,366**	**66**	**212,707**	**100**

© 2018 Giannakeas V et al. *JAMA Network Open.*

eTable 2. Matched DCIS patients treated with lumpectomy alone versus lumpectomy and radiation

Value	Lumpectomy alone	Lumpectomy and radiation	Standardized difference
Number of patients	29,465 (50.0%)	29,465 (50.0%)	
Year of diagnosis			
1998-2004	11,446 (38.8%)	11,446 (38.8%)	0
2005-2009	9,128 (31.0%)	9,128 (31.0%)	0
2010-2014	8,891 (30.2%)	8,891 (30.2%)	0
Age at diagnosis			
Mean (SD)	60.4 (11.8)	60.4 (11.8)	0
Median (IQR)	60.0 (51.0-70.0)	60.0 (51.0-69.0)	
Ethnicity			
White	23,473 (79.7%)	23,440 (79.6%)	0
Black	3,041 (10.3%)	3,043 (10.3%)	0
East Asian	1,155 (3.9%)	1,212 (4.1%)	0.01
Southeast Asian	1,057 (3.6%)	1,024 (3.5%)	0.01
Other/Unknown	739 (2.5%)	746 (2.5%)	0
Annual household income, $			
Mean (SD)	35,522.1 (8,103.4)	35,425.7 (8,092.8)	0.01
Median (IQR)	34,970.0 (30,340.0-40,610.0)	35,020.0 (29,710.0-40,610.0)	
Tumour grade			
I	4,639 (15.7%)	4,639 (15.7%)	0
II	11,569 (39.3%)	11,569 (39.3%)	0
III/IV	7,807 (26.5%)	7,807 (26.5%)	0
Unknown	5,450 (18.5%)	5,450 (18.5%)	0
Tumour size			
Mean (SD)	1.2 (1.6)	1.3 (1.3)	0.01
Median (IQR)	0.8 (0.4-1.5)	0.9 (0.5-1.5)	
ER status			
Negative	1,538 (5.2%)	1,538 (5.2%)	0
Positive	15,368 (52.2%)	15,368 (52.2%)	0
Unknown	12,559 (42.6%)	12,559 (42.6%)	0
PR status			
Negative	2,854 (9.7%)	2,934 (10.0%)	0.01
Positive	12,966 (44.0%)	12,983 (44.1%)	0
Unknown	13,645 (46.3%)	13,548 (46.0%)	0.01

© 2018 Giannakeas V et al. *JAMA Network Open.*

eTable 3. Matched DCIS patients treated with lumpectomy alone versus mastectomy

Value	Lumpectomy alone	Mastectomy	Standardized difference
Number of patients	20,832 (50.0%)	20,832 (50.0%)	
Year of diagnosis			
1998-2004	8,115 (39.0%)	8,115 (39.0%)	0
2005-2009	6,531 (31.4%)	6,531 (31.4%)	0
2010-2014	6,186 (29.7%)	6,186 (29.7%)	0
Age at diagnosis			
Mean (SD)	59.1 (12.0)	59.0 (12.1)	0
Median (IQR)	58.0 (50.0-68.0)	58.0 (49.0-68.0)	
Ethnicity			
White	16,299 (78.2%)	16,262 (78.1%)	0
Black	2,211 (10.6%)	2,248 (10.8%)	0.01
East Asian	866 (4.2%)	843 (4.0%)	0.01
Southeast Asian	747 (3.6%)	798 (3.8%)	0.01
Other/Unknown	709 (3.4%)	681 (3.3%)	0.01
Annual household income, $			
Mean (SD)	35,211.0 (8,236.8)	35,075.1 (8,228.1)	0.02
Median (IQR)	34,970.0 (30,150.0-40,610.0)	34,970.0 (29,460.0-40,610.0)	
Tumour grade			
I	2,312 (11.1%)	2,312 (11.1%)	0
II	7,918 (38.0%)	7,918 (38.0%)	0
III/IV	6,761 (32.5%)	6,761 (32.5%)	0
Unknown	3,841 (18.4%)	3,841 (18.4%)	0
Tumour size			
Mean (SD)	1.5 (2.1)	1.6 (1.8)	0.04
Median (IQR)	1.1 (0.5-2.0)	1.2 (0.6-2.0)	
ER status			
Negative	1,242 (6.0%)	1,242 (6.0%)	0
Positive	10,672 (51.2%)	10,672 (51.2%)	0
Unknown	8,918 (42.8%)	8,918 (42.8%)	0
PR status			
Negative	2,248 (10.8%)	2,361 (11.3%)	0.02
Positive	8,911 (42.8%)	8,818 (42.3%)	0.01
Unknown	9,673 (46.4%)	9,653 (46.3%)	0

© 2018 Giannakeas V et al. *JAMA Network Open.*

eTable 4. Matched DCIS patients treated with lumpectomy and radiation versus mastectomy

Value	Lumpectomy and radiation	Mastectomy	Standardized difference
Number of patients	29,865 (50.0%)	29,865 (50.0%)	
Year of diagnosis			
1998-2004	10,217 (34.2%)	10,217 (34.2%)	0
2005-2009	9,460 (31.7%)	9,460 (31.7%)	0
2010-2014	10,188 (34.1%)	10,188 (34.1%)	0
Age at diagnosis			
Mean (SD)	57.1 (11.3)	57.0 (11.3)	0
Median (IQR)	56.0 (48.0-65.0)	56.0 (48.0-65.0)	
Ethnicity			
White	23,238 (77.8%)	23,152 (77.5%)	0.01
Black	3,214 (10.8%)	3,227 (10.8%)	0
East Asian	1,277 (4.3%)	1,317 (4.4%)	0.01
Southeast Asian	1,216 (4.1%)	1,227 (4.1%)	0
Other/Unknown	920 (3.1%)	942 (3.2%)	0
Annual household income, $			
Mean (SD)	34,909.9 (8,346.4)	34,850.1 (8,368.5)	0.01
Median (IQR)	34,970.0 (28,200.0-40,610.0)	34,970.0 (28,500.0-40,610.0)	
Tumour grade			
I	2,441 (8.2%)	2,441 (8.2%)	0
II	9,957 (33.3%)	9,957 (33.3%)	0
III/IV	13,167 (44.1%)	13,167 (44.1%)	0
Unknown	4,300 (14.4%)	4,300 (14.4%)	0
Tumour size			
Mean (SD)	1.7 (1.6)	1.7 (1.6)	0.04
Median (IQR)	1.3 (0.7-2.1)	1.5 (0.8-2.2)	
ER status			
Negative	2,970 (9.9%)	2,970 (9.9%)	0
Positive	16,521 (55.3%)	16,521 (55.3%)	0
Unknown	10,374 (34.7%)	10,374 (34.7%)	0
PR status			
Negative	4,782 (16.0%)	4,846 (16.2%)	0.01
Positive	13,554 (45.4%)	13,480 (45.1%)	0
Unknown	11,529 (38.6%)	11,539 (38.6%)	0

© 2018 Giannakeas V et al. *JAMA Network Open.*

eTable 5. Breast cancer-specific mortality rates from DCIS diagnosis for the entire cohort and according to treatment group (mastectomy, lumpectomy alone, lumpectomy and radiotherapy)

Year	Complete DCIS Cohort				Mastectomy				Lumpectomy and radiation				Lumpectomy alone			
	FU time (years)	Events	Ann. Rate (%)	Cum. Rate (%)	FU time (years)	Events	Rate (%)	Cum. Rate (%)	FU time (years)	Events	Rate (%)	Cum. Rate (%)	FU time (years)	Events	Rate (%)	Cum. Rate (%)
**Overall**	986,201	1,104	0.112	-	280,835	359	0.128	-	452,365	403	0.089	-	253,001	342	0.135	-
0 - 1	134,429	45	0.033	0.033	38,270	16	0.042	0.042	62,614	14	0.022	0.022	33,545	15	0.045	0.045
1 - 2	123,463	74	0.060	0.093	35,046	28	0.080	0.122	57,508	22	0.038	0.061	30,909	24	0.078	0.122
2 - 3	113,060	85	0.075	0.168	32,036	24	0.075	0.197	52,592	29	0.055	0.116	28,431	32	0.113	0.235
3 - 4	102,368	109	0.106	0.275	28,986	34	0.117	0.314	47,514	38	0.080	0.196	25,868	37	0.143	0.378
4 - 5	91,818	119	0.130	0.404	25,965	35	0.135	0.448	42,487	41	0.097	0.292	23,366	43	0.184	0.561
5 - 6	81,451	113	0.139	0.542	22,936	44	0.192	0.639	37,666	38	0.101	0.393	20,849	31	0.149	0.710
6 - 7	70,964	97	0.137	0.678	19,931	34	0.171	0.808	32,753	35	0.107	0.500	18,280	28	0.153	0.863
7 - 8	61,225	100	0.163	0.840	17,218	33	0.192	0.999	28,021	40	0.143	0.642	15,986	27	0.169	1.031
8 - 9	52,022	87	0.167	1.006	14,737	30	0.204	1.200	23,522	31	0.132	0.774	13,763	26	0.189	1.220
9 - 10	43,694	70	0.160	1.165	12,508	18	0.144	1.342	19,489	32	0.164	0.938	11,696	20	0.171	1.391
10 - 11	35,629	64	0.180	1.342	10,318	17	0.165	1.505	15,704	29	0.185	1.122	9,606	18	0.187	1.578
11 - 12	28,051	49	0.175	1.515	8,258	19	0.230	1.731	12,175	20	0.164	1.286	7,619	10	0.131	1.709
12 - 13	20,879	42	0.201	1.713	6,298	10	0.159	1.887	8,903	17	0.191	1.477	5,677	15	0.264	1.973
13 - 14	14,078	23	0.163	1.873	4,256	11	0.258	2.141	5,949	7	0.118	1.594	3,873	5	0.129	2.101
14 - 15	8,031	13	0.162	**2.032**	2,481	3	0.121	**2.259**	3,338	5	0.150	**1.744**	2,213	5	0.226	**2.327**
15 - 16	3,894	13	0.334	2.359	1,215	2	0.165	2.420	1,656	5	0.302	2.045	1,022	6	0.587	2.913
16 - 17	1,145	1	0.087	2.445	376	1	0.266	2.680	473	-	-	2.045	296	-	-	2.913

Abbreviations: Ann., annual; Cum., cumulative

© 2018 Giannakeas V et al. *JAMA Network Open.*

eTable 6. Hazard ratios associated with radiation/extent of surgery using multivariate cox regression, inverse probability treatment weighting and 1:1 propensity score-based matching, with and without accounting for competing risks of death among matched subgroups

Comparison	Model 1: matched subgroups (non-competing risks model)		Model 2: matched subgroups (competing risks model)*		Model 3: inverse probability treatment weighting		Model 4: multivariate Cox regression**	
	Hazard Ratio (95% CI)	P-value	Hazard Ratio (95% CI)	P-value	Hazard Ratio (95% CI)	P-value	Hazard Ratio (95% CI)	P-value
Lumpectomy alone	1.0		1.0		1.0		1.0	
Lumpectomy + radiation	0.77 (0.67 - 0.88)	<.001	0.84 (0.74 – 0.96)	.008	0.79 (0.69 – 0.91)	.002	0.79 (0.68 – 0.92)	.002
Lumpectomy alone	1.0		1.0		1.0		1.0	
Mastectomy	0.91 (0.78 - 1.05)	.20	0.96 (0.83 – 1.10)	.52	0.90 (0.78 – 1.04)	.14	0.89 (0.76 – 1.04)	.15
Mastectomy	1.0		1.0		1.0		1.0	
Lumpectomy + radiation	0.75 (0.65 - 0.87)	<.001	0.79 (0.69 – 0.91)	<.001	0.78 (0.67 – 0.90)	<.001	0.81 (0.70 – 0.94)	.006

*Model considers other non-breast cancer-specific deaths as competing risks

**Adjusted for year of diagnosis (categorical), age at diagnosis (continuous), ethnicity, tumour grade, tumour size (continuous), ER-status and PR-status

© 2018 Giannakeas V et al. *JAMA Network Open.*

eTable 7. Breast cancer-specific mortality and ipsilateral invasive recurrence rates from DCIS diagnosis among matched DCIS patients treated with lumpectomy alone versus lumpectomy and radiation

eTable 7a. Breast cancer-specific mortality rates from DCIS diagnosis among matched DCIS patients treated with lumpectomy alone versus lumpectomy and radiation

Year	Lumpectomy and radiation				Lumpectomy alone			
	FU time (years)	Events	Rate (%)	Cumulative Rate (%)	FU time (years)	Events	Rate (%)	Cumulative Rate (%)
**Overall**	216,369	261	0.121	-	221,601	220	0.099	-
0 - 1	28,200	12	0.043	0.043	28,389	10	0.035	0.035
1 - 2	26,028	14	0.054	0.096	26,336	11	0.042	0.077
2 - 3	24,015	26	0.108	0.204	24,396	17	0.070	0.147
3 - 4	21,942	24	0.109	0.314	22,387	24	0.107	0.254
4 - 5	19,894	33	0.166	0.479	20,409	18	0.088	0.342
5 - 6	17,844	26	0.146	0.624	18,397	22	0.120	0.461
6 - 7	15,732	26	0.165	0.788	16,292	16	0.098	0.559
7 - 8	13,834	16	0.116	0.903	14,315	18	0.126	0.684
8 - 9	11,961	18	0.150	1.052	12,415	15	0.121	0.804
9 - 10	10,232	15	0.147	1.197	10,650	14	0.131	0.934
10 - 11	8,479	17	0.201	1.395	8,860	22	0.248	1.180
11 - 12	6,767	8	0.118	1.512	7,017	15	0.214	1.391
12 - 13	5,035	12	0.238	1.747	5,189	10	0.193	1.581
13 - 14	3,392	5	0.147	1.891	3,457	5	0.145	1.724
14 - 15	1,891	3	0.159	**2.047**	1,923	1	0.052	**1.775**
15 - 16	865	6	0.694	2.726	913	2	0.219	1.990
16 - 17	259	-	-	2.726	255	-	-	1.990

© 2018 Giannakeas V et al. *JAMA Network Open.*

eTable 7b. Ipsilateral invasive recurrence rates from DCIS diagnosis among matched DCIS patients treated with lumpectomy alone versus lumpectomy and radiation

Year	Lumpectomy and radiation				Lumpectomy alone			
	FU time (years)	Events	Rate (%)	Cumulative Rate (%)	FU time (years)	Events	Rate (%)	Cumulative Rate (%)
**Overall**	210,819	1,162	0.551	-	219,292	565	0.258	-
0 - 1	28,141	140	0.498	0.498	28,380	11	0.039	0.039
1 - 2	25,834	152	0.588	1.083	26,311	34	0.129	0.168
2 - 3	23,699	120	0.506	1.584	24,328	52	0.214	0.381
3 - 4	21,547	122	0.566	2.141	22,281	52	0.233	0.614
4 - 5	19,435	107	0.551	2.680	20,270	43	0.212	0.825
5 - 6	17,338	96	0.554	3.219	18,227	58	0.318	1.140
6 - 7	15,200	98	0.645	3.843	16,093	38	0.236	1.374
7 - 8	13,313	67	0.503	4.327	14,099	50	0.355	1.723
8 - 9	11,470	62	0.541	4.844	12,184	43	0.353	2.070
9 - 10	9,747	61	0.626	5.439	10,416	47	0.451	2.512
10 - 11	8,031	41	0.511	5.922	8,628	41	0.475	2.975
11 - 12	6,385	31	0.485	6.379	6,801	35	0.515	3.475
12 - 13	4,723	25	0.529	6.874	5,010	22	0.439	3.899
13 - 14	3,166	19	0.600	7.433	3,318	23	0.693	4.565
14 - 15	1,761	12	0.682	**8.064**	1,837	13	0.708	**5.240**
15 - 16	795	8	1.006	8.989	865	3	0.347	5.569
16 - 17	235	1	0.426	9.376	243	-	-	5.569

© 2018 Giannakeas V et al. *JAMA Network Open.*

eTable 8. Breast cancer-specific mortality and ipsilateral invasive recurrence rates from DCIS diagnosis among matched DCIS patients treated with mastectomy versus lumpectomy and radiation

eTable 8a. Breast cancer-specific mortality rates from DCIS diagnosis among matched DCIS patients treated with mastectomy versus lumpectomy and radiation

Year	Lumpectomy and radiation				Mastectomy			
	FU time (years)	Events	Rate (%)	Cumulative Rate (%)	FU time (years)	Events	Rate (%)	Cumulative Rate (%)
**Overall**	213,227	207	0.097	-	210,580	254	0.121	-
0 - 1	28,717	7	0.024	0.024	28,606	12	0.042	0.042
1 - 2	26,427	8	0.030	0.055	26,202	22	0.084	0.126
2 - 3	24,183	15	0.062	0.117	23,952	19	0.079	0.205
3 - 4	21,924	25	0.114	0.231	21,684	22	0.101	0.306
4 - 5	19,700	16	0.081	0.312	19,475	22	0.113	0.419
5 - 6	17,531	15	0.086	0.397	17,266	31	0.180	0.598
6 - 7	15,304	24	0.157	0.553	15,014	24	0.160	0.757
7 - 8	13,290	21	0.158	0.710	12,975	27	0.208	0.963
8 - 9	11,360	17	0.150	0.859	11,092	21	0.189	1.151
9 - 10	9,629	17	0.177	1.034	9,413	10	0.106	1.256
10 - 11	7,999	12	0.150	1.182	7,802	13	0.167	1.420
11 - 12	6,383	11	0.172	1.353	6,255	13	0.208	1.625
12 - 13	4,760	7	0.147	1.498	4,756	6	0.126	1.749
13 - 14	3,188	5	0.157	1.652	3,162	7	0.221	1.967
14 - 15	1,748	2	0.114	**1.765**	1,805	2	0.111	**2.075**
15 - 16	843	5	0.593	2.347	853	2	0.235	2.305
16 - 17	243	-	-	2.347	268	1	0.373	2.669

© 2018 Giannakeas V et al. *JAMA Network Open.*

eTable 8b. Ipsilateral invasive recurrence rates from DCIS diagnosis among matched DCIS patients treated with mastectomy versus lumpectomy and radiation

Year	Lumpectomy and radiation				Mastectomy			
	FU time (years)	Events	Rate (%)	Cumulative Rate (%)	FU time (years)	Events	Rate (%)	Cumulative Rate (%)
**Overall**	210,816	621	0.295	-	209,409	240	0.115	-
0 - 1	28,707	18	0.063	0.063	28,570	38	0.133	0.133
1 - 2	26,395	42	0.159	0.222	26,153	14	0.054	0.186
2 - 3	24,105	51	0.212	0.433	23,888	27	0.113	0.299
3 - 4	21,798	64	0.294	0.725	21,602	25	0.116	0.415
4 - 5	19,541	55	0.281	1.005	19,381	26	0.134	0.548
5 - 6	17,337	61	0.352	1.353	17,159	23	0.134	0.682
6 - 7	15,093	46	0.305	1.654	14,902	24	0.161	0.842
7 - 8	13,070	50	0.383	2.030	12,863	24	0.187	1.027
8 - 9	11,126	55	0.494	2.514	10,985	15	0.137	1.162
9 - 10	9,395	40	0.426	2.929	9,310	8	0.086	1.247
10 - 11	7,777	38	0.489	3.403	7,712	6	0.078	1.323
11 - 12	6,174	33	0.534	3.920	6,182	4	0.065	1.387
12 - 13	4,579	25	0.546	4.444	4,699	1	0.021	1.408
13 - 14	3,045	26	0.854	5.260	3,122	2	0.064	1.471
14 - 15	1,660	11	0.663	**5.888**	1,778	2	0.112	**1.582**
15 - 16	791	6	0.758	6.602	840	1	0.119	1.699
16 - 17	223	-	-	6.602	263	-	-	1.699

© 2018 Giannakeas V et al. *JAMA Network Open.*

eFigure 1. Breast cancer-specific survival post-DCIS in propensity-matched patients treated with lumpectomy alone vs. with mastectomy

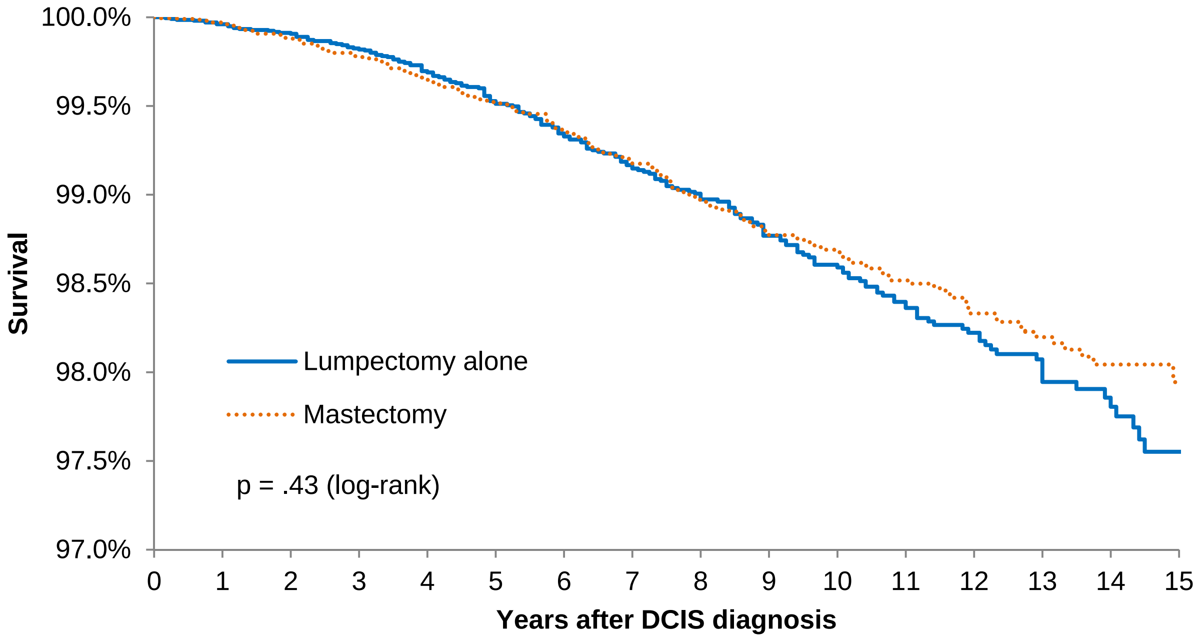

Number at risk	0	5	10	15
Lumpectomy alone	20,832	13,338	6,513	846
Mastectomy	20,832	13,556	6,655	857

© 2018 Giannakeas V et al. *JAMA Network Open.*

eFigure 2. Ipsilateral invasive recurrence-free survival post-DCIS in propensity-matched patients treated with lumpectomy and radiation vs. with lumpectomy alone

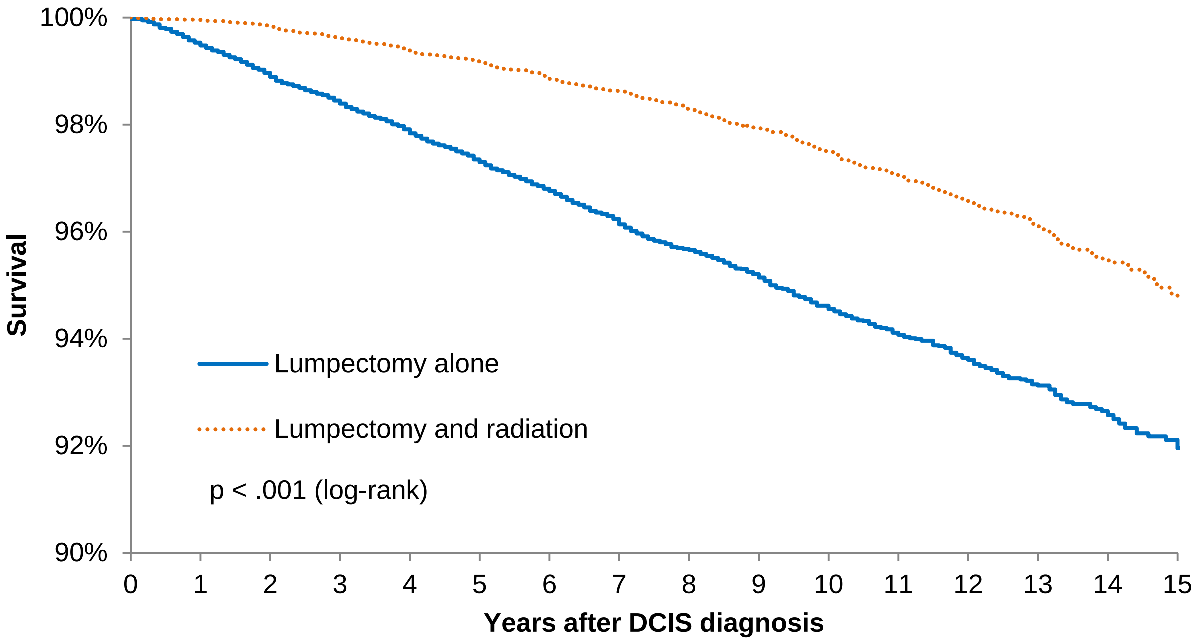

**Number at risk**	**0**	**5**	**10**	**15**
Lumpectomy alone	29,458	18,339	8,813	1,127
Lumpectomy and radiation	29,465	19,373	9,689	1,248

© 2018 Giannakeas V et al. *JAMA Network Open.*

eFigure 3. Breast cancer-specific survival post-DCIS in propensity-matched patients treated with lumpectomy and radiation vs. with mastectomy

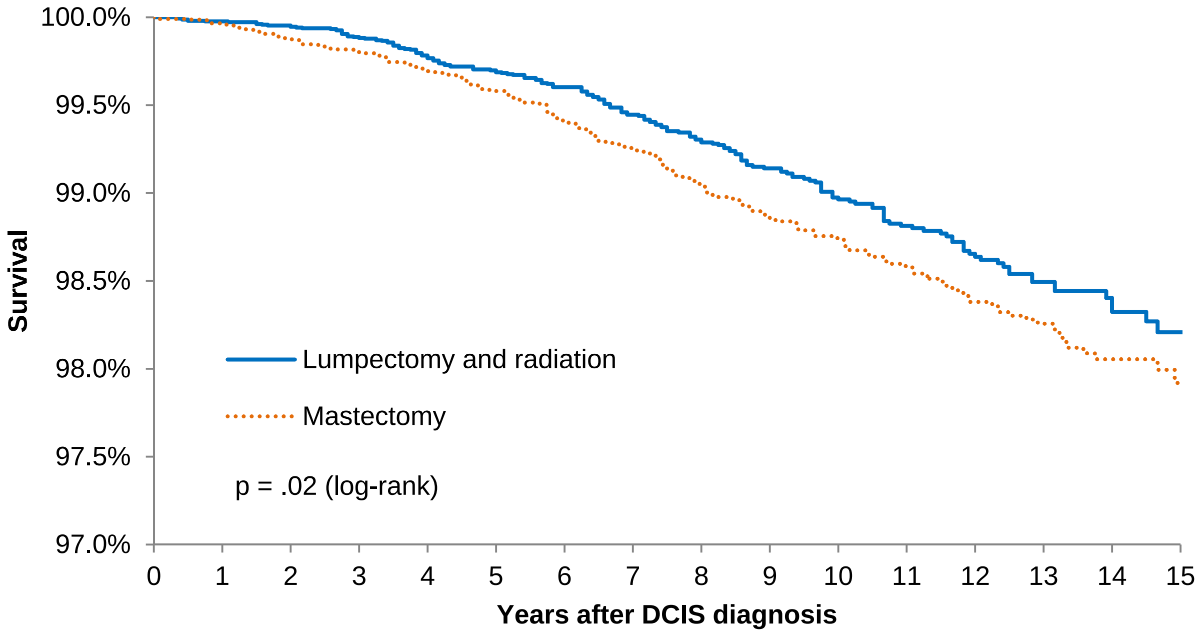

**Number at risk**	**0**	**5**	**10**	**15**
Lumpectomy and radiation	29,865	18,551	8,735	1,143
Mastectomy	29,865	18,311	8,562	1,131

© 2018 Giannakeas V et al. *JAMA Network Open.*

eFigure 4. Ipsilateral invasive recurrence-free survival post-DCIS in propensity-matched patients treated with lumpectomy and radiation vs. with mastectomy

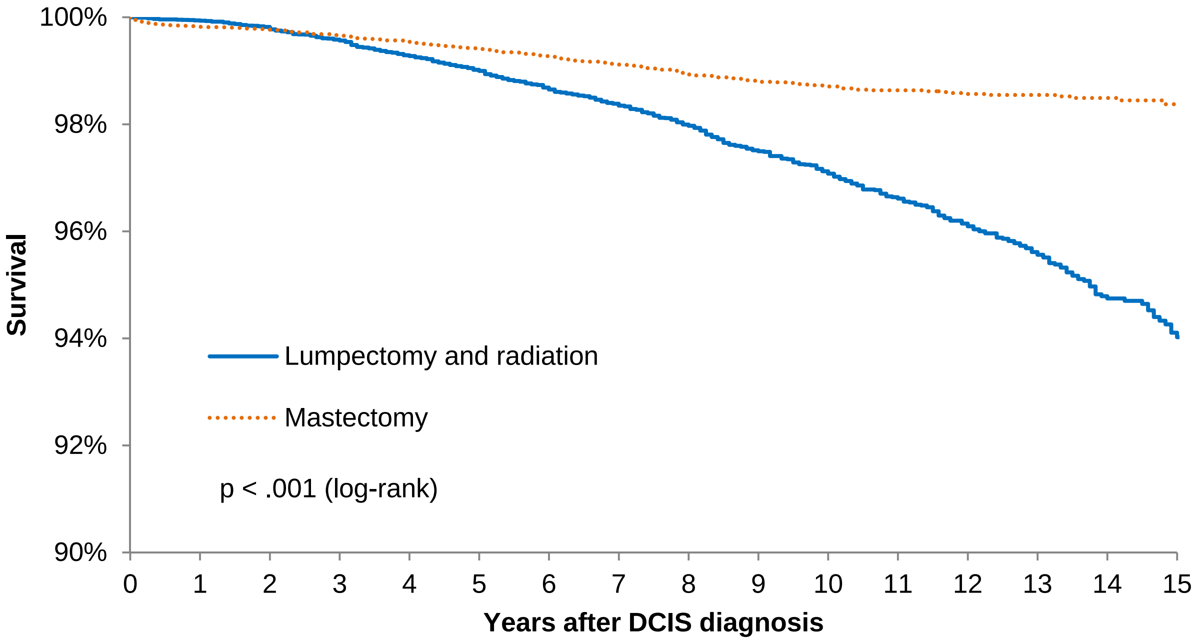

**Number at risk**	**0**	**5**	**10**	**15**
Lumpectomy and radiation	29,864	18,376	8,509	1,080
Mastectomy	29,850	18,209	8,463	1,113

© 2018 Giannakeas V et al. *JAMA Network Open.*

eFigure 5. Contralateral invasive breast cancer-free survival post-DCIS in propensity-matched patients treated with lumpectomy and radiation vs. with lumpectomy alone

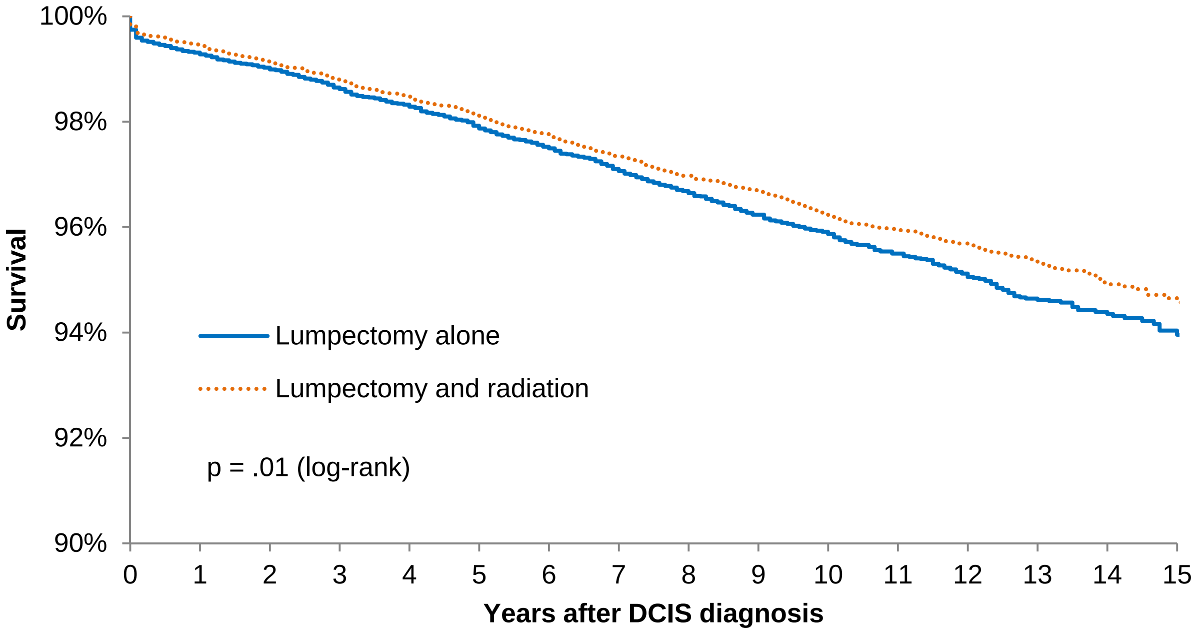

**Number at risk**	**0**	**5**	**10**	**15**
Lumpectomy alone	29,390	18,428	8,907	1,146
Lumpectomy and radiation	29,411	19,019	9,342	1,186

© 2018 Giannakeas V et al. *JAMA Network Open.*
